# Hippocampal NMDA receptors are important for behavioural inhibition but not for encoding associative spatial memories

**DOI:** 10.1098/rstb.2013.0149

**Published:** 2014-01-05

**Authors:** A. M. Taylor, T. Bus, R. Sprengel, P. H. Seeburg, J. N. P. Rawlins, D. M. Bannerman

**Affiliations:** 1 Department of Experimental Psychology, University of Oxford, South Parks Road, Oxford OX1 3UD, UK; 2 Max Planck Institute for Medical Research, Heidelberg 69120, Germany

**Keywords:** hippocampus, long-term potentiation, synaptic plasticity, spatial learning, watermaze, NMDAR

## Abstract

The idea that an NMDA receptor (NMDAR)-dependent long-term potentiation-like process in the hippocampus is the neural substrate for associative spatial learning and memory has proved to be extremely popular and influential. However, we recently reported that mice lacking NMDARs in dentate gyrus and CA1 hippocampal subfields (*GluN1*^*ΔDGCA1*^ mice) acquired the open field, spatial reference memory watermaze task as well as controls, a result that directly challenges this view. Here, we show that *GluN1*^*ΔDGCA1*^ mice were not impaired during acquisition of a spatial discrimination watermaze task, during which mice had to choose between two visually identical beacons, based on extramaze spatial cues, when all trials started at locations equidistant between the two beacons. They were subsequently impaired on test trials starting from close to the decoy beacon, conducted post-acquisition. *GluN1*^*ΔDGCA1*^ mice were also impaired during reversal of this spatial discrimination. Thus, contrary to the widely held belief, hippocampal NMDARs are not required for encoding associative, long-term spatial memories. Instead, hippocampal NMDARs, particularly in CA1, act as part of a comparator system to detect and resolve conflicts arising when two competing, behavioural response options are evoked concurrently, through activation of a behavioural inhibition system. These results have important implications for current theories of hippocampal function.

## 1. Introduction

The way in which associative memories are encoded and stored in the mammalian brain is one of the central questions in neuroscience. Hebb [1] suggested that associative memories are stored as changes in the strength or efficacy of the synaptic connections between neurons. The discovery of long-term potentiation (LTP) provided an experimental model of synaptic plasticity, of the type envisaged to underlie memory formation. It is now over 40 years since LTP was first reported by Bliss & Lomo [2], following experiments in the dentate gyrus (DG) subfield of the hippocampus in anaesthetized rabbits. Subsequently, the hypothesis that an LTP-like mechanism provides the neural basis for memory formation has gained considerable momentum. More specifically, the idea that NMDA receptor (NMDAR)-dependent LTP or an LTP-like process in the hippocampus (in particular in the CA1 subfield) is the neural substrate for associative spatial learning and memory has proved to be extremely popular and influential [3,4].

A cornerstone of this hypothesis is a series of papers by Tsien *et al*. [5,6], which describe a genetically modified mouse line in which they knocked out the obligatory GluN1 (formerly NR1) subunit of the NMDAR, and thus deleted NMDARs preferentially from the CA1 subfield of the hippocampus (T29-1 line). These mice lacked LTP at Schaffer collateral/commissural–CA1 synapses and were impaired on the standard, spatial reference memory version of the watermaze task. This was seen as a key result and it was taken as almost irrefutable proof that NMDAR-dependent LTP at CA1 synapses was indeed the neural substrate for associative, long-term spatial memory formation.

However, it was subsequently reported that Cre expression and NMDAR ablation extended beyond the hippocampus in these mice [7–11]. There is demonstrable Cre expression in cortical principal neurons and a reduction in cortical GluN1 expression in the T29-1 line. Thus, the conclusion that hippocampal CA1 NMDARs (and by inference NMDAR-dependent synaptic plasticity) underlie spatial reference memory acquisition in the watermaze is confounded by a potential extra-hippocampal contribution to the phenotype in these T29-1 GluN1 mice.

We recently reported on genetically modified mice which lack the obligatory GluN1 subunit, and hence NMDARs, in both the pyramidal cells of CA1 and the granule cells of the DG [12]. In these *GluN1*^*ΔDGCA1*^mice, Cre expression is fairly well restricted to these hippocampal principal cells, although there is some expression in olfactory bulb granule cells, and in a small number of layer II piriform cortex neurons. Thus, there is only minimal NMDAR ablation in cortex. As expected, no NMDAR-mediated responses could be evoked at CA3-to-CA1 synapses in dorsal hippocampal slices from adult *GluN1*^*ΔDGCA1*^ mice, and LTP could not be induced in the Schaffer collateral/commissural pathway. An unexpected side effect of the mutation in the *GluN1*^*ΔDGCA1*^ mice was an atrophy of the granule cell layer in the DG of these animals. Nevertheless, these mice provide an alternative tool for testing the hippocampal LTP/spatial memory hypothesis.

Strikingly, these *GluN1*^*ΔDGCA1*^ mice acquired the classic, open-field, spatial reference memory watermaze task as well as controls [12]. In fact, on the transfer (probe) test at the end of watermaze training, the knockouts actually spent more time searching in the training quadrant than the controls. The inclusion of a hippocampal lesion group confirmed that our watermaze paradigm was still hippocampus-dependent. Furthermore, the fact that the *GluN1*^*ΔDGCA1*^ mice appeared to outperform the controls during the transfer tests, often seen as the gold-standard measure of watermaze performance, rules out the possibility that the lack of a deficit was simply due to the task not being sensitive enough. Thus, these data suggested that hippocampal NMDARs are not in fact required for forming long-term, associative spatial memories.

However, the *GluN1*^*ΔDGCA1*^ mice were impaired when the platform was subsequently moved to the opposite quadrant of the pool during spatial reversal testing. They were also substantially impaired at acquiring the spatial reference memory component of the radial maze task, during which mice have to learn to discriminate between arms that always contain food rewards and arms that are never baited. Thus, hippocampal NMDARs clearly still play an important role in aspects of spatial memory performance, just not necessarily in the formation of associative, long-term spatial memories. This striking dissociation between spatial reference memory acquisition in the watermaze and spatial reference memory acquisition on the radial maze was not due to the different sensorimotor or motivational demands of these two classic spatial memory tasks. Instead, it reflected differences in the psychological processes involved.

To investigate this further, a subsequent set of watermaze experiments was conducted. Separate groups of experimentally naive *GluN1*^*ΔDGCA1*^ mice and controls were trained on either a spatial or a non-spatial discrimination task, using visible beacons to indicate the position of the platform. In the spatial version, mice were trained to discriminate between two visually identical beacons (black spheres sitting on the water surface), based on their allocentric spatial locations, as defined by the extramaze room cues, in order to locate the hidden escape platform. In the non-spatial version, two visually distinct beacons were used (a grey funnel versus a black/white-striped cylinder), and their spatial locations varied from trial to trial. In the non-spatial task, one visual stimulus was always associated with the escape platform, irrespective of its spatial location. *GluN1*^*ΔDGCA1*^ mice were significantly impaired on the spatial discrimination task but not on the non-spatial version. They made significantly more choice errors than controls on the spatial paradigm. Crucially, however, this did not reflect a lack of knowledge about where the escape platform was located. Using the classic transfer (or probe) tests, in which both of the beacons and the escape platform were removed from the pool, we showed that the *GluN1*^*ΔDGCA1*^ mice spent as much time searching in the training quadrant as the controls, demonstrating that they knew as much about the spatial location of the platform.

Thus, we argued that this was not a spatial learning deficit but rather it reflected an inability to use the spatial cues to behaviourally inhibit the very strong conditioned response to swim towards the first beacon that the mice encountered. A similar account could explain the impairment on the reference memory radial maze task, where the animals might use the extramaze spatial cues to inhibit the very strong conditioned response that they have acquired to run down any arm of the maze to obtain food rewards (see [12] for discussion). Importantly, the deficit on the watermaze discrimination task was specific to the situation that arose when the beacons were ambiguous (i.e. when two visually identical beacons were used). There was no deficit when there were two visually distinct, and hence unambiguous, beacons.

Consistent with this account, we showed that the increased number of choice errors in the *GluN1*^*ΔDGCA1*^ mice specifically reflected their performance on trials when they were started from a position at the edge of the pool which was in close proximity to the decoy (S−) beacon. We therefore argued that this was not a problem with memory acquisition or encoding but rather with behavioural inhibition. In this study, we have now tested this hypothesis further by training mice on the spatial discrimination watermaze task but with all trials starting from a location equidistant between the two beacons. Both control and *GluN1*^*ΔDGCA1*^ mice acquired this version of the task successfully, and at an equivalent rate. However, *GluN1*^*ΔDGCA1*^ mice were subsequently impaired on test trials that started from close to the decoy beacon, and that were conducted post-acquisition, consistent with our hypothesis. *GluN1*^*ΔDGCA1*^ mice were also impaired during reversal of the spatial discrimination task.

## 2. Methods

### (a) Subjects

Generation of the mice, and their subsequent histological and electrophysiological analysis has been described in detail previously [12]. Mice were on a C57BL/6N background. Animals were housed on a 12-h light–dark cycle (lights on at 07.00 and off at 19.00), with all testing conducted during the light phase. They had *ad libitum* access to food and water throughout. Mice were experimentally naive at the start of testing, which was conducted under the auspices of UK Home Office Project and Personal licences held by the authors. Mice were approximately eight to nine months of age at the start of testing. Control (male: *n* = 5, female: *n* = 5) and *GluN1*^*ΔDGCA1*^ mice (male: *n* = 6, female: *n* = 5) were compared. Preliminary inspection of the data revealed no obvious differences between male and female animals, and therefore for the purposes of analysis mice from both genders have been combined.

### (b) Apparatus

**Figure 1.**
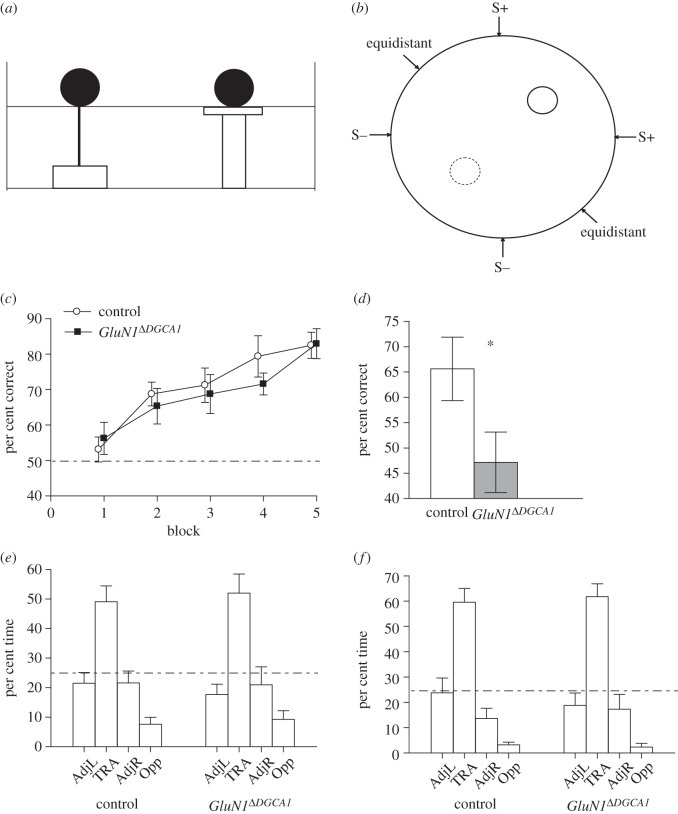
Acquisition of a spatial discrimination task in the Morris watermaze for control and *GluN1*^*ΔDGCA1*^ mice. (*a,b*) Mice were trained to find an escape platform (unbroken circle) by discriminating between two visually identical beacons (broken circle corresponds to the decoy beacon), depending on their spatial locations. During acquisition, mice were placed into the pool from either of the equidistant start locations. During subsequent testing (S− start test trials and reversal), mice were placed into the pool at one of six possible start positions. (*c*) Acquisition: both control (*n* = 10; white circles) and *GluN1*^*ΔDGCA1*^ mice (*n* = 11; black squares) learned to choose the correct beacon at an equivalent rate. Mean per cent correct choices ± s.e.m. for each block of 16 trials. (*d*) *GluN1*^*ΔDGCA1*^ mice (grey bar) were impaired at choosing the correct beacon relative to controls (white bar) when subsequent test trials started from close to the decoy beacon (S− start locations). Mean per cent correct choices ± s.e.m. for 16 S− start trials. Asterisk denotes significant genotype difference, *p* < 0.05. (*e,f*) Both control and *GluN1*^*ΔDGCA1*^ mice exhibited a strong preference for the training quadrant (TRA) during transfer test 1 and 2. Mean per cent time spent in each quadrant ± s.e.m. Broken line denotes chance levels of performance (25%).

Mice were trained to find an escape platform (20 cm diameter), using a spatial discrimination variant of the watermaze task (figure 1*a,b*). This study used the same watermaze (2 m diameter), in the same laboratory, with the same extramaze spatial cues as in our previous study [12]. The water temperature was maintained at 20 ± 1°C. In order to escape from the water the mice had to find a fixed location, hidden escape platform (diameter: 20 cm) submerged approximately 1 cm below the water surface. The platform was located at the centre of either the NE or SW quadrant of the pool (figure 1*b*). The number of mice trained to each platform position was counterbalanced with respect to group.

### (c) Pre-training

Mice were first trained to approach a single black, spherical, plastic beacon (diameter: 15 cm; height: 24 cm), sitting on the water surface, which indicated the position of the escape platform (eight trials per day for 3 days). The spatial location of the beacon and the start position of the animal changed from one trial to the next, according to a pseudorandom sequence. By the end of this stage of training, all mice were swimming directly to the beacon and escaping from the water onto the platform.

### (d) Spatial discrimination training

The mice were then trained to discriminate between two identical, visible beacons (black, spherical, plastic beacons), depending on their spatial locations. Both beacons remained in a fixed location in space throughout testing and were arranged in diametrically opposite quadrants of the pool (i.e. in the NE and SW quadrants). One beacon indicated the position of the escape platform (S+ beacon), whereas the other beacon was attached to a thin metal pole in order to hold it in a fixed position at the water's surface, but provided no means of escape from the water (S− beacon; figure 1*a*). The allocation of mice to a particular S+ beacon/platform location was counterbalanced with respect to genotype.

Mice were placed into the water facing the side wall from one of two possible start locations equidistant between the two beacons, according to a pseudorandom sequence (figure 1*b*; equidistant). For half of the trials the S+ beacon/platform was to the left of the start position and for half of the trials it was positioned on the right. Mice received 10 days of spatial discrimination training (eight trials per day).

On top of each beacon was a circular (20 cm diameter) piece of laminated white card. On the S+ beacon, this white circle sat exactly above the position of the escape platform. Mice were considered to have made an error and chosen the wrong option when they passed under the white circle on the S− beacon. Whether the first choice that the mouse made was correct or incorrect was recorded (choice accuracy: per cent correct). In addition, we also counted the total number of errors made on a given trial. For example, if a mouse swam under the S− beacon and then re-emerged before, again, swimming under the S− beacon, then this was scored as two errors.

In addition, we then also conducted a standard probe trial (transfer test 1) at the end of 10 days of acquisition training. This probe trial assessed the extent to which the mice had learned about the spatial location of the platform with respect to the extramaze room cues, and was exactly the same as the probe trials used with the standard, open-field, reference memory watermaze paradigm [12]. Both beacons and the escape platform were removed from the pool, and the mouse was allowed to swim freely for 60 s. The percentage of time that animals spent in each quadrant of the pool was recorded. The number of annulus crossings (swim paths over the former platform location and analogous positions in the other three quadrants) were also recorded. The probe trial was conducted 24 h after the last training trial. Mice were then given one further day of acquisition training (with equidistant start positions as described earlier) to counter any possible extinction that might have occurred as a result of the transfer test.

### (e) Test trials starting from close to the decoy (S−) beacon

After the mice had acquired the task, they were then given a series of test trials during which they were started from a position at the perimeter of the pool close to the decoy (S−) beacon (figure 1*b*). Mice received a total of 16 trials from the S− start position. For symmetry with the subsequent reversal phase (see below), mice also received trials starting from close to the correct (S+) beacon and further trials starting from a point equidistant between the two beacons. Thus, mice were now tested from multiple start locations around the perimeter of the pool. They were placed into the water facing the side wall from one of six possible start locations according to a pseudorandom sequence. For half of the trials the S+ beacon/platform was on the left and for half of the trials, it was positioned on the right. Two of the start positions were equidistant between the two beacons, two were closer to the S+ beacon/platform (approx. 80 cm from the platform), and two were closer to the S− beacon (i.e. 140 cm from the platform; figure 1*b*). Mice received eight trials per day for a total of 6 days. Therefore, over 6 days of testing each mouse received eight trials from each of the six different start positions (i.e. 16 trials from S− starts, 16 trials from equidistant starts and 16 trials from S+ starts). They received no more than three consecutive trials from the same start position. A second transfer test (transfer test 2) was then conducted after the completion of these S− test trials. Again, both beacons and the escape platform were removed from the pool and the mouse was allowed to swim freely for 60 s.

### (f) Reversal

Reversal of the spatial discrimination was then assessed. The escape platform was now moved to the opposite quadrant of the pool (e.g. NE to SW; SW to NE). It was still associated with one of the black spherical beacons but now it was located in the diametrically opposite spatial location. Mice were again placed into the water facing the side wall from one of six possible start locations, according to a pseudorandom sequence (figure 1*b*). For half of the trials, the S+ beacon/platform was on the left, and for half of the trials, it was positioned on the right. In addition, two of the start positions were equidistant between the two beacons, two were closer to the S+ beacon/platform and two were closer to the S− beacon. Mice received eight trials per day for 12 days with the platform in its new spatial location.

## 3. Results

**Figure 2.**
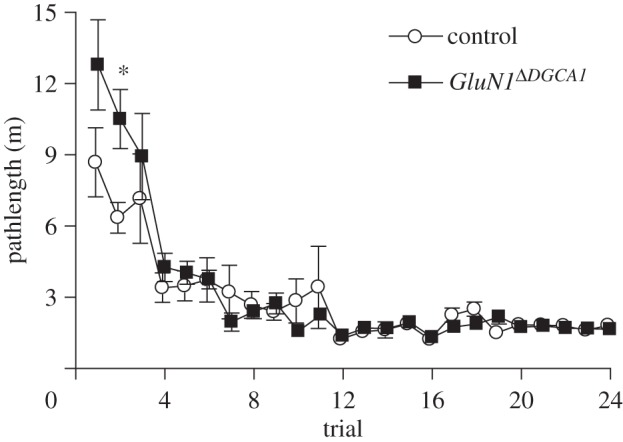
Performance of control (*n* = 10; white circles) and *GluN1*^*ΔDGCA1*^ mice (*n* = 11; black squares) during non-spatial pre-training with a single visible beacon/platform (variable platform location and variable start location). Mean pathlength (m) ± s.e.m. for each trial. Asterisk denotes significant genotype difference, *p* = 0.01.

During non-spatial pre-training with a single beacon (variable start location and variable platform location), all mice rapidly learned to swim directly to the beacon and escape from the water by climbing onto the platform. Analysis of pathlengths to escape to the platform revealed a main effect of trial (*F*_23,437_ = 25.37; *p* < 0.0001), but no overall main effect of genotype (*F*_1,19_ = 1.28; *p* > 0.20; figure 2). There was, however, a significant genotype by trial interaction (*F*_23,437_ = 1.91; *p* < 0.010), which reflected the prolonged pathlengths of the *GluN1*^*ΔDGCA1*^ mice on the first two trials, suggesting a possibly abnormal response to the novelty of the situation in these animals. Analysis of simple main effects revealed that there was a significant effect of genotype on trial 2 only (*F*_1,19_ = 8.29; *p* = 0.01). On trial 3, the pathlengths of the two groups were not different, and control and *GluN1*^*ΔDGCA1*^ mice then continued to perform equivalently throughout the rest of pre-training.

Mice were then trained on the spatial discrimination task with two visually identical beacons, with all trials starting from either of the equidistant start locations (figure 1*c*: acquisition). Both groups acquired the task at an equivalent rate, and there was no sign of any impairment in the *GluN1*^*ΔDGCA1*^ mice. Analysis of first choice accuracy (per cent correct trials) revealed a significant main effect of block (*F*_4,76_ = 17.25; *p* < 0.0001), which reflected the learning of the task. However, there was no main effect of genotype, nor genotype by block interaction (both *F* < 1; *p* > 0.60). Analysis of total errors showed a very similar outcome. There was a significant main effect of block (*F*_4,76_ = 19.97; *p* < 0.0001), reflecting the reduction in errors as the animals learned, but again there was no main effect of genotype (*F*_1,19_ = 1.54; *p* > 0.20), and no genotype by block interaction (*F* < 1; *p* > 0.90; data not shown).

To assess further the extent to which the mice had learned about the spatial location of the platform, a transfer test was performed during which the escape platform and both beacons were removed from the pool and the mice allowed to swim freely for 60 s (figure 1*e*; transfer test 1). Both groups spent most of their time searching in the quadrant of the pool that normally contained the escape platform. This preference for the training quadrant (TRA) was equivalent in control and *GluN1*^*ΔDGCA1*^ mice. ANOVA revealed a main effect of quadrant (*F*_2,57_ = 23.06; *p* < 0.05; note that for the analysis of the distribution of time spent searching the four quadrants, the numerator term in the degrees of freedom was reduced by one to control for the fact that the quadrant dwell times were not independent), but no genotype by quadrant interaction (*F* < 1; *p* > 0.90). A separate analysis of time spent in the training quadrant only also revealed no group difference (*t* < 1; *p* > 0.70). Annulus crossings data revealed a similar pattern of results. There was a significant main effect of quadrant (*F*_3,57_ = 18.12; *p* < 0.0001), reflecting the preference of the mice for the training quadrant, but no significant effect of genotype (*F*_1,19_ = 2.01; *p* > 0.10), and no genotype by quadrant interaction (*F* < 1; *p* > 0.60; data not shown).

Mice then received one further day of acquisition training before then receiving test trials during which they were placed into the pool at a point close to the decoy beacon (S− start locations; figure 1*b*). The *GluN1*^*ΔDGCA1*^ mice were now impaired at choosing successfully between the two beacons (figure 1*d*). Analysis of first choice accuracy revealed a significant deficit in the knockout animals (*t*_19_ = 2.13; *p* < 0.05). Inspection of the total number of errors made on these S− start trials also revealed a marginally significant difference between the two groups (*t*_19_ = 2.09; *p* = 0.050; data not shown).

A second transfer test was then conducted (transfer test 2; figure 1*f*), which again demonstrated that both groups had a strong and equivalent preference for the training quadrant, both in terms of time spent (main effect of quadrant; *F*_2,57_ = 44.18; *p* < 0.0001, genotype by quadrant interaction; *F* < 1; *p* > 0.80), and in terms of annulus crossings (main effect of quadrant; *F*_3,57_ = 27.60; *p* < 0.0001, no effect of genotype; *F*_1,19_ = 1.10; *p* > 0.30, and no genotype by quadrant interaction; *F* < 1; *p* > 0.60, data not shown). A separate comparison of time spent in the training quadrant only confirmed the lack of a genotype difference (*t* < 1; *p* > 0.70).

**Figure 3.**
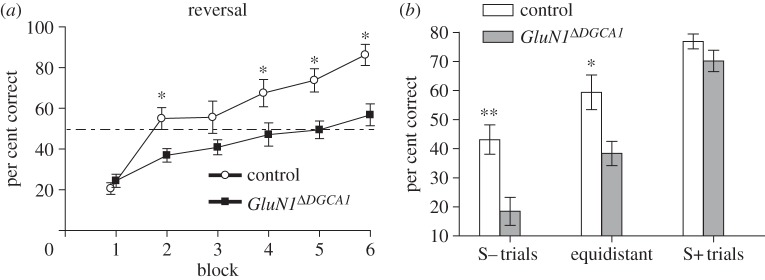
Reversal of the spatial discrimination in control and *GluN1*^*ΔDGCA1*^ mice. (*a*) *GluN1*^*ΔDGCA1*^ mice (*n* = 11; black squares) were impaired compared with controls (*n* = 10; white circles) during reversal in terms of first choice accuracy. Mean per cent correct choices ± s.e.m. for each block of 16 trials. Asterisk denotes significant genotype difference, *p* < 0.05. (*b*) First choice accuracy for controls (white bars) and *GluN1*^*ΔDGCA1*^ mice (grey bars) on trials starting from close to the decoy beacon (S− trials), or equidistant, or close to the correct beacon (S+ trials). Mean per cent correct choices ± s.e.m. collapsed across 32 trials for each start position type. Asterisks denote significant genotype difference, *p < 0.01, **p < 0.005.

The spatial location of the escape platform was then switched to the diametrically opposite quadrant of the pool, although it was still associated with one of the beacons (reversal). There was now a dramatic impairment in the *GluN1*^*ΔDGCA1*^ mice (figure 3). Comparison of first choice accuracy (per cent correct trials) for control and *GluN1*^*ΔDGCA1*^ mice revealed a highly significant main effect of genotype (*F*_1,19_ = 12.04; *p* < 0.005), a significant main effect of block which reflected reversal learning across blocks (*F*_5,95_ = 34.56; *p* < 0.0001), and a significant genotype by block interaction (*F*_5,95_ = 4.01; *p* < 0.005; figure 3*a*). Subsequent analysis of simple main effects showed that the two groups performed equivalently on the first block of reversal testing (*F* < 1; *p* > 0.30), but then the *GluN1*^*ΔDGCA1*^ mice were significantly impaired on blocks 2, 4, 5 and 6 (*F*_1,19_ ≥ 5.43; *p* < 0.05; the group difference on block 3 just failed to attain statistical significance; *F*_1,19_ = 3.01; *p* < 0.10). Analysis of total errors also revealed a substantial impairment in the *GluN1*^*ΔDGCA1*^ mice during spatial reversal (main effect of genotype; *F*_1,19_ = 11.42; *p* < 0.005, main effect of block; *F*_5,95_ = 45.59; *p* < 0.0001, and no genotype by block interaction; *F*_5,95_ = 1.54; *p* > 0.10; data not shown).

We also re-analysed the reversal data in terms of the starting position of the trials. This analysis suggested that the deficit was greatest on trials starting from close to the decoy beacon (S− start positions) and least on trials starting from close to the correct beacon (S+ trials; figure 3*b*). ANOVA comparing first choice accuracy on trials from each of the three possible start positions revealed an overall effect of start position (*F*_2,38_ = 78.88; *p* < 0.0001), reflecting the fact that performance was best on S+ trials and worst on S− trials, and a significant genotype by start location interaction (*F*_2,38_ = 3.86; *p* < 0.05). Subsequent analysis of simple main effects showed that there were significant genotype differences on S− start trials (*F*_1,19_ = 12.43; *p* < 0.005), and, importantly, also on equidistant start trials (*F*_1,19_ = 8.66; *p* < 0.01), but not on S+ start trials (*F*_1,19_ = 2.15; *p* > 0.10). The significant reversal impairment on equidistant start trials is in contrast to the lack of an effect during the original acquisition of the spatial discrimination task with equidistant start trials. A similar analysis for total errors also showed an overall effect of start location (*F*_2,38_ = 32.48; *p* < 0.0001), but the genotype by start location interaction just failed to reach statistical significance (*F*_2,38_ = 2.99; *p* = 0.06, data not shown).

## 4. Discussion

Mice lacking NMDARs in both CA1 pyramidal cells and DG granule cells successfully acquired a spatial discrimination version of the Morris watermaze task when all trials were started at locations equidistant between the two beacons, performing as well as controls. This was evident both in terms of the accuracy of their choice behaviour (first choice accuracy), and also in terms of their preference for the training quadrant, measured in two transfer tests during which the beacons and the escape platform were removed from the pool and the mice allowed to swim freely for 60 s. These data replicate our previous findings with these *GluN1*^*ΔDGCA1*^ mice [12], demonstrating that mice without NMDARs in CA1, and hence lacking LTP at Schaffer collateral/commissural pathway synapses, can acquire long-term, associative spatial memories perfectly well.

The performance of *GluN1*^*ΔDGCA1*^ mice was, however, subsequently impaired on test trials starting from close to the decoy beacon (S− trials), which were conducted post-acquisition, after the animals had already acquired the spatial discrimination task successfully. This result adds further support to our previous suggestion that the impairments that these mice show on spatial memory tasks do not reflect a problem with the acquisition or encoding of associative spatial memories, but instead they reflect an inability to use the extramaze spatial cues to behaviourally inhibit the very strong conditioned response to swim towards the first beacon that the mice encounter. Of course, this effect will be maximized on trials starting from close to the decoy beacon.

In addition, the *GluN1*^*ΔDGCA1*^ mice were also dramatically impaired during reversal of the spatial discrimination task, when the escape platform was subsequently moved to the opposite quadrant of the pool (figure 3). This reversal deficit was hugely significant on trials starting from close to the decoy beacon (S− trials), but, importantly, it was also highly significant on equidistant start trials. This is in marked contrast to the clear lack of impairment observed for acquisition of the spatial discrimination task in this study, during which all trials were started from points equidistant between the two beacons (figure 1).

Thus, *GluN1*^*ΔDGCA1*^ mice are not impaired at forming long-term, associative spatial memories, but do display a deficit on the spatial discrimination beacon task when trials are started from close to the decoy beacon, and are also impaired during spatial reversal learning. We will now compare these data with earlier studies in rats using the NMDAR antagonist D-AP5. In fact, there is actually remarkably good correspondence between much of this pharmacological data and the results obtained with *GluN1*^*ΔDGCA1*^ mice. Hence, our results are far from unprecedented.

### (a) Hippocampal NMDARs are not required for forming long-term, associative spatial memories

Although our results appear to be at odds with the original watermaze deficit reported with the NMDAR antagonist D-AP5 [13,14], they are, to a large extent, consistent with subsequent pharmacological studies using D-AP5, which showed, somewhat surprisingly, that if animals received prior spatial pre-training as normal animals (i.e. in the absence of any drug), and were then tested in the presence of D-AP5 (30 mM) in a completely novel spatial environment (i.e. a completely different laboratory), then the NMDAR antagonist was now found to have little, if any, effect on acquisition of the open-field, spatial reference memory task (the spatial upstairs/downstairs task [15]; see also [16,17]). Therefore, these pharmacological studies also demonstrated that hippocampal NMDARs are not required for (i) spatial navigation *per se*, (ii) forming a cognitive map of a novel environment, nor (iii) forming a long-term association between a particular spatial location and an outcome (e.g. an escape platform). Therefore, these AP5 data also challenge the popular hypothesis that NMDAR-dependent synaptic plasticity at CA1 synapses is the neural substrate for associative, long-term spatial memory formation.

### (b) A role for hippocampal NMDARs in spatial discrimination performance

Notably, D-AP5-treated rats *are* impaired on the spatial discrimination version of the watermaze task, during which the animals have to choose between two visually identical platforms, only one of which provides an escape from the water [18]. In fact, this deficit is observed even if spatial pre-training is given prior to drug infusion, in contrast to the upstairs/downstairs open-field spatial memory task [19]. It is also worth pointing out that in this rat study, D-AP5 impaired acquisition of the spatial discrimination task despite the fact that all trials were started from locations that were equidistant between the two beacons, in contrast to the results with *GluN1*^*ΔDGCA1*^ mice in this study (figure 1*c*). These different outcomes could reflect the potentially more widespread effect on NMDAR function following drug infusion, compared with our regionally selective knockout mice. Alternatively, it could reflect important species differences between rats and mice, which may be related to the relative size of the animals and/or the way that they behave on being placed in the water. Nevertheless, both D-AP5-treated rats and *GluN1*^*ΔDGCA1*^ mice display impairments during performance of the spatial discrimination task.

### (c) A role for hippocampal NMDARs during spatial reversal

*GluN1*^*ΔDGCA1*^ mice were also dramatically impaired during reversal of the spatial discrimination task. This deficit does not simply reflect a general problem with reversal learning *per se*. As previously mentioned, in our earlier study, we also compared *GluN1*^*ΔDGCA1*^ mice and controls on a non-spatial, visual discrimination version of the watermaze task, in which mice had to choose between two visually distinct beacons (a black/white-striped cylinder and a grey funnel). Not only did the *GluN1*^*ΔDGCA1*^ mice acquire this non-spatial beacon task perfectly well (see fig. 7 of [12]), but they were also completely unimpaired during its subsequent reversal when the platform was then switched to being associated with the opposite visual cue (see fig. 10 in supplementary information of [12]). It is of course tempting to attribute these different outcomes to the spatial/non-spatial nature of the two discrimination tasks, given the prominent role in spatial information processing attributed to the hippocampus. However, it is also important to point out that these *GluN1*^*ΔDGCA1*^ mice are perfectly capable of very high levels of spatial memory performance (figure 1). In fact, their performance on the standard, open-field version of the Morris watermaze task was as good, if not better, than that of the controls (see fig. 3 of [12]).

Thus, although spatial reversal is exquisitely sensitive to hippocampal NMDAR ablation, this is not the case for spatial acquisition. We have observed a similar pattern of results with *GluN1*^*ΔDGCA1*^ mice in the classic, open-field version of the watermaze task. As previously mentioned, *GluN1*^*ΔDGCA1*^ mice acquired the standard, fixed location, hidden escape platform version of the watermaze task as well as controls but were then substantially impaired during a subsequent spatial reversal (see fig. 3 of [12]).

There is also a precedent that spatial reversal tasks are more sensitive to NMDAR disruption than de novo spatial acquisition from the earlier pharmacological studies with D-AP5. Morris *et al*. [20] showed that rats treated with D-AP5 (30 mM) were impaired on a spatial reversal task in the open-field watermaze. In this study, the rats were pre-trained as normal animals on the standard spatial reference memory version of the watermaze task to find a hidden escape platform in a fixed spatial location. Testing then continued in the presence of AP5 or aCSF, but now the platform was moved to the diametrically opposite quadrant of the pool (e.g. from NE to SW). The AP5-treated rats were slower to search at the new platform location, demonstrating impaired spatial reversal. Notably, this result contrasts with the outcome of the upstairs/downstairs watermaze experiments which were conducted with D-AP5 in the same laboratory, but which revealed no impairment with the drug [15]. Crucially, rats used in the spatial reversal task and rats used in the upstairs/downstairs task received exactly the same amount and pattern of spatial pre-training, and subsequently received the same dose of 30 mM D-AP5, thus allowing direct comparison across studies. It is notable that pre-trained, AP5-treated animals were impaired on the spatial reversal version of the task but not on the spatial upstairs/downstairs task. Thus, the requirement for NMDAR-dependent synaptic plasticity is greater when the animal has to learn a new goal location within a familiar environment, compared with learning an entirely new spatial layout. Neither the original hippocampal LTP/spatial memory hypothesis [4,14], nor a sensorimotor account [21,22] or spatial strategy account [15] can adequately explain this AP5 dataset.

### (d) An alternative role for hippocampal NMDARs

We suggest that hippocampal NMDARs are not required for the acquisition or storage of associative spatial memories. Instead, they play a key role in using spatial or contextual information to disambiguate and select between competing and conflicting memories, and the resulting behavioural response choices that they support [12]. Historically, the idea of the hippocampus as a key component of a comparator system which can detect mismatch or conflict is a very old one, which predates even the cognitive map hypothesis [23–25]. There is evidence from a variety of sources that implicates the hippocampus, and particularly the CA1 subfield, with some form of comparator function, and with the ability to detect and/or resolve associative mismatch and conflict [26–32]. This could involve comparing an expectation based on information retrieved from long-term memory to information about the current state of the perceptual world. The comparator could also be activated when there is a requirement to disambiguate between competing or overlapping long-term memories, in order to prevent inappropriately cued or conditioned responses being made. It is important to point out that the purpose of this hippocampal comparator is *not* as a reward prediction error signal which will determine the extent of associative learning [33,34]. Instead, the consequences of activating the hippocampal comparator system are behavioural inhibition of on-going motor activity, coupled with increases in attention and arousal levels. Consequently, this allows within-trial modification of performance without affecting the progressive changes in associative learning that occur from trial to trial.

Such an account clearly contrasts with more traditional, memory encoding accounts of hippocampal NMDAR function, including not only the original hippocampal LTP/spatial memory hypothesis but also more recent derivatives from this idea which have emphasized the rapid and/or automatic encoding of associative memories [35]. For example, it might be argued that the deficits seen in both the *GluN1*^*ΔDGCA1*^ mice and the AP5-treated rats on the spatial reversal tasks (including the delayed match to place watermaze task which could be considered as a daily sequence of new spatial reversals [36,37]), could reflect a more rapid and/or automatic form of encoding, compared with the more gradual acquisition observed during the standard, spatial reference memory watermaze task.

However, it is difficult to conceive how either (i) the impairment seen in the *GluN1*^*ΔDGCA1*^ mice on the spatial reference memory component of the radial maze task, or (ii) the previous demonstration of impaired spatial discrimination watermaze task performance (using multiple start positions; see [12] for both tasks), could be due to a problem with the rapid encoding of associative spatial memories. Both these tasks are acquired gradually. In the case of the spatial discrimination watermaze task, acquisition of the discrimination occurs at a parallel rate to learning about the relationship between the platform location and the extramaze room cues. It is not obvious why learning about the relationship between the beacon, the escape platform and the extramaze room cues would recruit a rapid encoding mechanism, whereas learning about the relationship between just the escape platform and the extramaze room cues would not. It is also important to point out that the deficit in the *GluN1*^*ΔDGCA1*^ mice on the spatial discrimination task was dependent on their starting position in the pool, relative to the two ambiguous beacons. The first choice accuracy of the *GluN1*^*ΔDGCA1*^ mice during acquisition of the spatial discrimination task was only significantly disrupted on trials starting from close to the decoy beacon (S− trials; see fig. 6*e* of [12]). Furthermore, in this study, we have gone one step further and demonstrated that the *GluN1*^*ΔDGCA1*^ mice exhibit impaired performance on S− start trials, having previously acquired that task perfectly well from equidistant start locations. In our view, this does not reflect a memory encoding problem but rather it is a problem with recruiting a behavioural inhibition system when faced with ambiguous or uncertain cues.

There are a number of possible neural substrates for the behavioural phenotype that we see in the *GluN1*^*ΔDGCA1*^ mice. As reported previously [12], these mice lack NMDARs in both the CA1 pyramidal cells and DG granule cells. In addition, there is also a reduction in granule cell numbers. Any of these effects, alone or in combination, could be responsible for the behavioural impairments. Therefore, the effects on spatial discrimination performance, spatial reversal and acquisition of the spatial reference memory component of the radial maze, observed in this study and in our earlier work, could be due to ablation of NMDARs on DG granule cells and/or the atrophy of the granule cells. Against this, however, it is worth pointing out that acquisition of the spatial reference memory radial maze task was not impaired when the NMDAR knockout was limited to the DG granule cells only [38], in contrast to the result in the *GluN1*^*ΔDGCA1*^ mice [12]. This might suggest that it is the ablation of NMDARs from CA1 pyramidal cells that is responsible for the behavioural deficits, although it could also reflect a cumulative effect in both CA1 and DG. It is also possible that the behavioural phenotype reflects the absence of an NMDAR-dependent plasticity process in these cells, other than LTP (e.g. a long-term depression-like plasticity [39]), or some other NMDAR-dependent mechanism altogether. An important question is for how long the behavioural consequences of activating the hippocampal comparator system (e.g. behavioural inhibition of on-going motor activity, increases in attention and arousal) actually last. This will be a key consideration when searching for potential neural substrates.

## 5. Conclusion

To conclude, contrary to the widely held belief, hippocampal NMDARs are not required for forming associative, long-term spatial memories. Instead, we argue that hippocampal NMDARs, and particularly those in the CA1 subfield, might act as part of a comparator system to detect and resolve conflicts arising when two competing, behavioural response options are evoked concurrently, through subsequent activation of a behavioural inhibition system. This role for hippocampal NMDARs is borne out of an alternative account of hippocampal function which attempts to explain not only the role of the dorsal hippocampus in spatial memory but also the distinct role of the ventral subregion in anxiety [25,40,41].

Furthermore, we suggest that it is not the NMDAR-dependent LTP/spatial memory hypothesis that is wrong. This hypothesis could still very well be correct but with extra-hippocampal NMDARs as its substrate. After all, there is considerable evidence that NMDARs in the forebrain *do* make an essential contribution on spatial reference memory tasks such as the watermaze [6,10,14,42,43]. Indeed, it is almost impossible to imagine that NMDARs and NMDAR-dependent synaptic plasticity are not important for spatial learning and memory. Instead, we argue that it is the role of the hippocampus that has been misunderstood and needs to be reconsidered.

## References

[1] Hebb DO 1949 The organization of behavior. New York, NY: John Wiley.

[2] Bliss TV Lomo T 1973 Long-lasting potentiation of synaptic transmission in the dentate area of the anaesthetized rabbit following stimulation of the perforant path. J. Physiol. 232, 331–356. 4727084 10.1113/jphysiol.1973.sp010273 PMC1350458

[3] Bliss TV Collingridge GL 1993 A synaptic model of memory: long-term potentiation in the hippocampus. Nature 361, 31–39. (10.1038/361031a0) 8421494

[4] Martin SJ Grimwood PD Morris RG 2000 Synaptic plasticity and memory: an evaluation of the hypothesis. Annu. Rev. Neurosci. 23, 649–711. (10.1146/annurev.neuro.23.1.649) 10845078

[5] Tsien JZ Chen DF Gerber D Tom C Mercer EH Anderson DJ Mayford M Kandel ER Tonegawa S 1996 Subregion- and cell type-restricted gene knockout in mouse brain. Cell 87, 1317–1326. (10.1016/S0092-8674(00)81826-7) 8980237

[6] Tsien JZ Huerta PT Tonegawa S 1996 The essential role of hippocampal CA1 NMDA receptor-dependent synaptic plasticity in spatial memory. Cell 87, 1327–1338. (10.1016/S0092-8674(00)81827-9) 8980238

[7] Wiltgen BJ 2010 A role for calcium-permeable AMPA receptors in synaptic plasticity and learning. PLoS ONE 5, e12818 (10.1371/journal.pone.0012818) 20927382 PMC2947514

[8] Hoeffer CA 2008 Removal of FKBP12 enhances mTOR–Raptor interactions, LTP, memory, and perseverative/repetitive behavior. Neuron 60, 832–845. (10.1016/j.neuron.2008.09.037) 19081378 PMC2630531

[9] Fukaya M Kato A Lovett C Tonegawa S Watanabe M 2003 Retention of NMDA receptor NR2 subunits in the lumen of endoplasmic reticulum in targeted NR1 knockout mice. Proc. Natl Acad. Sci. USA 100, 4855–4860. (10.1073/pnas.0830996100) 12676993 PMC153645

[10] Brigman JL 2010 Loss of GluN2B-containing NMDA receptors in CA1 hippocampus and cortex impairs long-term depression, reduces dendritic spine density, and disrupts learning. J. Neurosci. 30, 4590–4600. (10.1523/JNEUROSCI.0640-10.2010) 20357110 PMC2869199

[11] Rondi-Reig L 2006 Impaired sequential egocentric and allocentric memories in forebrain-specific-NMDA receptor knock-out mice during a new task dissociating strategies of navigation. J. Neurosci. 26, 4071–4081. (10.1523/JNEUROSCI.3408-05.2006) 16611824 PMC6673881

[12] Bannerman DM 2012 Dissecting spatial knowledge from spatial choice by hippocampal NMDA receptor deletion. Nat. Neurosci. 15, 1153–1159. (10.1038/nn.3166) 22797694 PMC3442238

[13] Morris RG 1989 Synaptic plasticity and learning: selective impairment of learning rats and blockade of long-term potentiation in vivo by the *N*-methyl-d-aspartate receptor antagonist AP5. J. Neurosci. 9, 3040–3057. 2552039 10.1523/JNEUROSCI.09-09-03040.1989 PMC6569656

[14] Morris RG Anderson E Lynch GS Baudry M 1986 Selective impairment of learning and blockade of long-term potentiation by an *N*-methyl-d-aspartate receptor antagonist, AP5. Nature 319, 774–776. (10.1038/319774a0) 2869411

[15] Bannerman DM Good MA Butcher SP Ramsay M Morris RGM 1995 Distinct components of spatial learning revealed by prior training and NMDA receptor blockade. Nature 378, 182–186. (10.1038/378182a0) 7477320

[16] Saucier D Cain DP 1995 Spatial learning without NMDA receptor-dependent long-term potentiation. Nature 378, 186–189. (10.1038/378186a0) 7477321

[17] Inglis J Martin SJ Morris RG 2013 Upstairs/downstairs revisited: spatial pretraining-induced rescue of normal spatial learning during selective blockade of hippocampal *N*-methyl-d-aspartate receptors. Eur. J. Neurosci. 37, 718–727. (10.1111/ejn.12087) 23278867

[18] Morris RG Steele RJ Bell JE Martin SJ 2013 *N*-methyl-d-aspartate receptors, learning and memory: chronic intraventricular infusion of the NMDA receptor antagonist d-AP5 interacts directly with the neural mechanisms of spatial learning. Eur. J. Neurosci. 37, 700–717. (10.1111/ejn.12086) 23311352

[19] Uekita T Okaichi H 2009 Pretraining does not ameliorate spatial learning deficits induced by intrahippocampal infusion of AP5. Behav. Neurosci. 123, 520–526. (10.1037/a0015672) 19485557

[20] Morris RG Davis S Butcher SP 1990 Hippocampal synaptic plasticity and NMDA receptors: a role in information storage? Phil. Trans. R. Soc. Lond. B 329, 187–204. (10.1098/rstb.1990.0164) 1978364

[21] Keith JR Rudy JW 1990 Why NMDA receptor-dependent long-term potentiation may not be a mechanism of learning and memory: re-appraisal of the NMDA receptor blockade strategy. Psychobiology 18, 251–257.

[22] Cain DP Saucier D Hall J Hargreaves EL Boon F 1996 Detailed behavioral analysis of water maze acquisition under APV or CNQX: contribution of sensorimotor disturbances to drug-induced acquisition deficits. Behav. Neurosci. 110, 86–102. (10.1037/0735-7044.110.1.86) 8652076

[23] Vinogradova OS 1975 Functional organisation of the limbic system in the process of registration of information: facts and hypotheses. In The hippocampus (ed. Pribram RIIKH), pp. 3–69. New York, NY: Plenum Press.

[24] Gray JA 1982 The neuropsychology of anxiety, 1st edn Oxford, UK: Oxford University Press.

[25] Gray JA McNaughton N 2000 The neuropsychology of anxiety, 2nd ed Oxford, UK: Oxford University Press.

[26] Ploghaus A Tracey I Clare S Gati JS Rawlins JNP Matthews PM 2000 Learning about pain: the neural substrate of the prediction error for aversive events. Proc. Natl Acad. Sci. USA 97, 9281–9286. (10.1073/pnas.160266497) 10908676 PMC16859

[27] Kumaran D Maguire EA 2007 Match mismatch processes underlie human hippocampal responses to associative novelty. J. Neurosci. 27, 8517–8524. (10.1523/JNEUROSCI.1677-07.2007) 17687029 PMC2572808

[28] Kumaran D Maguire EA 2006 An unexpected sequence of events: mismatch detection in the human hippocampus. PLoS Biol. 4, e424 (10.1371/journal.pbio.0040424) 17132050 PMC1661685

[29] O'Keefe J Nadel L 1978 The hippocampus as a cognitive map. Oxford, UK: Clarendon Press.

[30] O'Keefe J 1976 Place units in the hippocampus of the freely moving rat. Exp. Neurol. 51, 78–109. (10.1016/0014-4886(76)90055-8) 1261644

[31] Fyhn M Molden S Hollup S Moser M-B Moser EI 2002 Hippocampal neurons responding to first-time dislocation of a target object. Neuron 35, 555–566. (10.1016/S0896-6273(02)00784-5) 12165476

[32] Honey RC Watt A Good M 1998 Hippocampal lesions disrupt an associative mismatch process. J. Neurosci. 18, 2226–2230. 9482806 10.1523/JNEUROSCI.18-06-02226.1998 PMC6792925

[33] Hollerman JR Schultz W 1998 Dopamine neurons report an error in the temporal prediction of reward during learning. Nat. Neurosci. 1, 304–309. (10.1038/1124) 10195164

[34] Lisman JE Grace AA 2005 The hippocampal-VTA loop: controlling the entry of information into long-term memory. Neuron 46, 703–713. (10.1016/j.neuron.2005.05.002) 15924857

[35] Morris RG 2006 Elements of a neurobiological theory of hippocampal function: the role of synaptic plasticity, synaptic tagging and schemas. Eur. J. Neurosci. 23, 2829–2846. (10.1111/j.1460-9568.2006.04888.x) 16819972

[36] Nakazawa K Sun LD Quirk MC Rondi-Reig L Wilson MA Tonegawa S 2003 Hippocampal CA3 NMDA receptors are crucial for memory acquisition of one-time experience. Neuron 38, 305–315. (10.1016/S0896-6273(03)00165-X) 12718863

[37] Steele RJ Morris RG 1999 Delay-dependent impairment of a matching-to-place task with chronic and intrahippocampal infusion of the NMDA-antagonist D-AP5. Hippocampus 9, 118–136. (10.1002/(SICI)1098-1063(1999)9:2<118::AID-HIPO4>3.0.CO;2-8) 10226773

[38] Niewoehner B Single FN Hvalby Ã̃ Jensen V Meyer zum Alten Borgloh S Seeburg PH Rawlins JNP Sprengel R Bannerman DM 2007 Impaired spatial working memory but spared spatial reference memory following functional loss of NMDA receptors in the dentate gyrus. Eur. J. Neurosci. 25, 837–846. (10.1111/j.1460-9568.2007.05312.x) 17313573 PMC2777262

[39] Kemp A Manahan-Vaughan D 2007 Hippocampal long-term depression: master or minion in declarative memory processes? Trends Neurosci. 30, 111–118. (10.1016/j.tins.2007.01.002) 17234277

[40] Bannerman DM Rawlins JNP McHugh SB Deacon RMJ Yee BK Bast T Zhang W-N Pothuizen HHJ Feldon J 2004 Regional dissociations within the hippocampus—memory and anxiety. Neurosci. Biobehav. Rev. 28, 273–283. (10.1016/j.neubiorev.2004.03.004) 15225971

[41] Barkus C McHugh SB Sprengel R Seeburg PH Rawlins JNP Bannerman DM 2010 Hippocampal NMDA receptors and anxiety: at the interface between cognition and emotion. Eur. J. Pharmacol. 626, 49–56. (10.1016/j.ejphar.2009.10.014) 19836379 PMC2824088

[42] von Engelhardt J 2008 Contribution of hippocampal and extra-hippocampal NR2B-containing NMDA receptors to performance on spatial learning tasks. Neuron 60, 846–860. (10.1016/j.neuron.2008.09.039) 19081379

[43] Barkus C Dawson LA Sharp T Bannerman DM 2012 GluN1 hypomorph mice exhibit wide-ranging behavioral alterations. Genes Brain Behav. 11, 342–351. (10.1111/j.1601-183X.2012.00767.x) 22300668 PMC3489048

